# AFEAP cloning: a precise and efficient method for large DNA sequence assembly

**DOI:** 10.1186/s12896-017-0394-x

**Published:** 2017-11-14

**Authors:** Fanli Zeng, Jinping Zang, Suhua Zhang, Zhimin Hao, Jingao Dong, Yibin Lin

**Affiliations:** 1 College of Life Sciences, Hebei Agricultural University, Baoding, 071001 People’s Republic of China; 2 Institute of Biophysics, Hebei University of Technology, Tianjin, 300401 People’s Republic of China; 3 McGovern Medical School, the University of Texas Health Science Center at Houston, Houston, 77030 USA

**Keywords:** Synthetic biology, DNA assembly, Assembly of fragment ends after PCR, Multi-fragment assembly, Bacterial artificial chromosomes

## Abstract

**Background:**

Recent development of DNA assembly technologies has spurred myriad advances in synthetic biology, but new tools are always required for complicated scenarios. Here, we have developed an alternative DNA assembly method named AFEAP cloning (Assembly of Fragment Ends After PCR), which allows scarless, modular, and reliable construction of biological pathways and circuits from basic genetic parts.

**Methods:**

The AFEAP method requires two-round of PCRs followed by ligation of the sticky ends of DNA fragments. The first PCR yields linear DNA fragments and is followed by a second asymmetric (one primer) PCR and subsequent annealing that inserts overlapping overhangs at both sides of each DNA fragment. The overlapping overhangs of the neighboring DNA fragments annealed and the nick was sealed by T4 DNA ligase, followed by bacterial transformation to yield the desired plasmids.

**Results:**

We characterized the capability and limitations of new developed AFEAP cloning and demonstrated its application to assemble DNA with varying scenarios. Under the optimized conditions, AFEAP cloning allows assembly of an 8 kb plasmid from 1-13 fragments with high accuracy (between 80 and 100%), and 8.0, 11.6, 19.6, 28, and 35.6 kb plasmids from five fragments at 91.67, 91.67, 88.33, 86.33, and 81.67% fidelity, respectively. AFEAP cloning also is capable to construct bacterial artificial chromosome (BAC, 200 kb) with a fidelity of 46.7%.

**Conclusions:**

AFEAP cloning provides a powerful, efficient, seamless, and sequence-independent DNA assembly tool for multiple fragments up to 13 and large DNA up to 200 kb that expands synthetic biologist’s toolbox.

**Electronic supplementary material:**

The online version of this article (doi: 10.1186/s12896-017-0394-x) contains supplementary material, which is available to authorized users.

## Background

DNA sequence assembly, which refers to the precise aligning and merging multiple fragments of DNA, in an end-to-end fashion, into large synthetic circuits and pathways, plays a pivotal role in protein structure-function, metabolic engineering, and synthetic biology [1–5]. The increasingly high demand for assembling large DNA into functional devices requires the methods that allow scarless, sequence independent, multi-fragment assembly of large constructs at high efficiency and high fidelity [6, 7]. In the past decade, many novel DNA assembly methods, such as: Gibson Assembly (GA) [8], Golden Gate assembly [9], uracil-specific excision reagent cloning (USER) [10], ligase cycling reaction (LCR) [11], DNA assembler [12], twin-primer assembly (TPA) [6], sequence and ligation-independent cloning (SLIC) [13], seamless ligation cloning extract (SliCE) [14], enzyme-free cloning (EFC) [15], polymerase incomplete primer extension (PIPE) [16], in Vivo assembly (IVA cloning) [17], DNA assembly with thermostable exonuclease and ligase (DATEL) [7], and overlap extension PCR and recombination (OEPR Cloning) [18], have been designed and developed (Additional file 1: Table S1), which opened doors to a wide variety of applications. These methods differ in both mechanism and scale, providing the effective means to cope with different needs [2, 4]. Recent advances in synthetic biology would be aided by these new techniques [4]. Despite the advantage of these assembly techniques, to our knowledge, no one approach can satisfy all even most of the requirements and each still has its limitations. As such, the development of novel, easy-to-use, scarless assembly methods with high efficiency and accuracy, especially for multiple fragments and large DNA, are always required [7].

In the present study, inspired by the concept of restriction-free cloning method [19] and recent advances in high fidelity DNA polymerase, such as G-HiFi™ DNA polymerase (up to 40 kb DNA with fidelity), we have designed and developed a novel, simple and robust protocol for the construction of large biochemical pathways, circuits, and plasmids. This system requires two rounds of PCRs to generate DNA fragments with compatible 5′ cohesive ends for scarless assembly of multiple DNA fragments with large size into a transformable plasmid. Since the system requires two rounds of PCRs followed by ligation of the sticky ends of DNA fragments, we named the method AFEAP cloning (Assembly of Fragment Ends After PCR). With this “hand in hand” cloning, constructions of an 8 kb plasmid from 1 to 12 fragments, four plasmids with varying sizes of 11.6, 19.6, 28, and 35.6 kb, and a 200 kb of bacterial artificial chromosome (BAC) were achieved with high fidelity.

## Results and discussions

### Overview of AFEAP cloning method

**Fig. 1:**
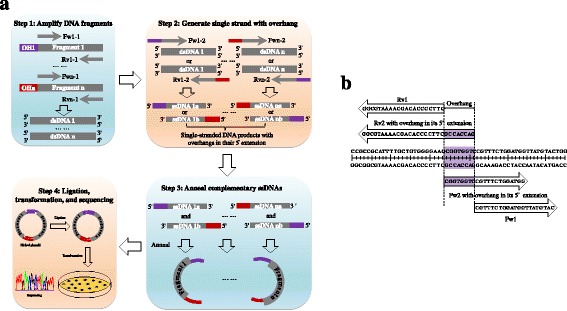
AFEAP cloning. **a** Schematic details show the flow chart of multi-fragment assembly with AFEAP cloning. The first PCR yields linear DNA fragments (step 1), and is followed by a second asymmetric (one primer) PCR (step 2) and subsequent annealing (step 3) that inserts overlapping overhangs at 5′ end of each DNA fragment. These double-stranded DNA fragments are then subsequently assembled “hand-in-hand” (step 4). The nicks in the annealed multi-part DNAs are sealed by DNA ligase to form transformable plasmid (step 4), followed by bacterial transformation to yield the desired plasmids, which is confirmed by DNA sequencing (step 4). **b** A typical AFEAP cloning showing the region of overhang, and the two set primers of two adjacent fragments. Fw: forward primer, Rv: reverse primer, OH: overhang region, ssDNA: single-stranded DNA

The mechanism of AFEAP cloning for assembling multiple fragments is shown in Fig. 1a. AFEAP cloning requires two-round of PCRs to generate overhang adapter sequence at 5′ ends of each DNA molecule that can associate to link DNA segments. All of the nicks between two adjacent fragments are joined by the T4 DNA ligase without the introduction of any scar sequences. The crucial point for successful AFEAP cloning is to assign an “overhang” region. As shown in Fig. 1a and b, the overhang can be a short sequence on the 5′ terminus of the joining sites. AFEAP cloning requires two sets of primers (Fig. 1b). The primers of the first set are designed standard forward and reverse primers that flank the assigned overhang region. The primers of the second set are designed that have additional overhang sequence at their 5′ ends that will then be incorporated into the PCR product. In detail, the assembly of DNA fragments with AFEAP cloning into circular plasmid requires four steps (Fig. 1a): (i) In the first-round PCR, several PCRs are carried out in parallel with forward and reverse primers of the first set, i.e., Fw1–1 and Rv1–1, Fw2–1 and Rv2–1, Fw3–1 and Rv3–1,…, and Fwn-1 and Rvn-1, to produce double-stranded DNA fragments, i.e., dsDNA 1, dsDNA 2, dsDNA 3,…, dsDNA n; (ii) In the second-round PCR, two single-primer PCRs run in parallel with each one of the forward and reverse primers of the second set, i.e., Fw1–2 or Rv1–2, Fw2–2 or Rv2–2, Fw3–2 or Rv3–2,…, or Fwn-2 or Rvn-2, using each DNA product generated in the first-round PCR as template. Second-round PCRs yield several pairs of complementary single-stranded DNA products that contain the desired overhang regions at their 5′ ends, i.e., ssDNA1a/ssDNA 1b, ssDNA 2a/ssDNA 2b, ssDNA 3a/ssDNA 3b,…, or ssDNA na/ssDNA nb; (iii) the complementary single-stranded DNA products generated in step 2 anneal to form double-stranded DNA fragments with 5′ unpaired overhang; (iv) These double-stranded DNA fragments are then subsequently assembled “hand-in-hand”. The nicks in the annealed multi-part DNAs are sealed by ligase to form transformable plasmid. Reconstituted vectors are transformed into competent *E.coli* cells and the joining sites can be confirmed by DNA sequencing.

### Determination of parameters for effective assembly

**Fig. 2:**
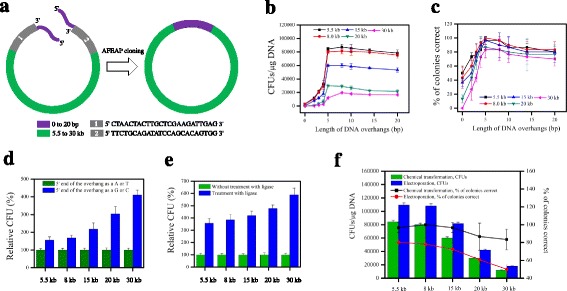
Determination of parameters for effective assembly. **a** Flowsheet of the assembly of 3′ and 5′ ends of linear DNA fragment to form the circle. Effects of the overhang length on the assembly efficiency were characterized as colony-forming units (CFUs) per microgram of ligated DNA (**b**) and percent of colonies correct (fidelity) (**c**). **d** Effects of the overhang designed as 5′ end of G/C or A/T. The relative CFUs produced of overhang designed as 5′ end of G or C are presented as percentages of the CFUs of overhang designed as 5′ end of A or T at the same construct size. **e** Effects of the ligase treated. The relative CFUs produced of ligase treated are presented as percentages of the CFUs of no ligase treated at the same construct size. **f** Effects of transformation conditions

To determine the optimum conditions for AFEAP cloning, we evaluated the effects of five key factors, such as overhang length, DNA fragments size, overhang designed as 5′ end of G/C or A/T, ligase treatment, and transformation conditions, which we had hypothesized to be important for AFEAP cloning. A set of DNA fragments with varying sizes (Fig. 2a) were used to evaluate the reaction system and its assembly efficiency. Primers designed for assembling 3′ and 5′ ends of linear DNAs to form the circle were listed in Additional file 2: Table S2. PCR products were subjected to AFEAP cloning protocol as mentioned above (Fig. 1a). The assembly efficiency of AFEAP cloning was characterized as colony-forming units (CFUs) per microgram of ligated DNA after transformation and the percentage of clones containing the desired vectors over total sequenced ones was calculated as fidelity. The join sites of each assembly were confirmed by DNA sequencing (Additional file 3: Figure S1).

We first tested the effects of the overhang length. The tested DNA overhangs length ranges from 0 to 20 bp. The overhang length was showed marked effects on assembly efficiency (Fig. 2b and c). An overhang, which is less than 2 nucleotides in the PCR products is insufficient for assembly, thereby resulting in low positive clones. From 4 nucleotides overhang onwards, a sharp increase of the efficiency of AFEAP cloning is observed up to 10 bp, with the efficiency peak at 9000 CFUs and 98% of colonies correct. From 10 nucleotides overhang onwards, longer overhangs used somehow decrease the efficiency slightly. As a result, 5–8 nucleotides overhang is, therefore, suitable for AFEAP cloning with high efficiency and low cost. And then we investigated the effects of the size of DNA fragments. Five different size points, i.e., 5.5, 8.0, 15, 20, and 30 kb were tested. The CFU did decrease significantly with longer DNA size fragments (Fig. 2b), while the fidelity did not change significantly within the length of overhang range tested (Fig. 2c). Moreover, we evaluated the effects of the overhangs designed as 5′ end of G/C or A/T. The assembly efficiency of DNA fragments, specifically for those of longer DNA size fragments, is benefiting from 5′ end of the overhang as a G or C (Fig. 2d). In addition, we tested the effects of the ligase treatment. As shown in Fig. 2e, the assembly efficiency for different size fragments did increase significantly when treated with ligase. Last, we evaluated the effects of transformation conditions, such as electroporation or chemical transformation, on the assemble efficiency. Electroporation gave higher efficiencies, but lower fidelities (Fig. 2f).

The optimal conditions for effective DNA assembly with AFEAP cloning were summarized and listed in Table 1.

**Table 1:** Summary of optimal conditions for DNA assembly with AFEAP cloning

Parameters	Tested conditions	Optimal conditions
Overhang length	ranges from 0 to 20 nucleotides	5–8 nucleotides
Size of DNA fragments	5.5, 8.0, 15, 20, and 30 kb	Decreased with the increase of DNA size
Overhangs designed as 5′ end of G/C or A/T	overhangs designed as 5′ end of G/C or A/T	G/C
Ligase treatment	T4 DNA ligase treated or not	Ligase treated
Ligation ratio (Shorter to longer)	1:6; 1:3; 1:1; 3:1; 6:1; 10:1, 15:1; and 20:1	10:1
Transformation conditions	Chemical, electroporation	Chemical transformation
DNA fragment number	2, 3, 4, 5, 6, 7, 8, 9, 10, 11, and 12	Decreased with the increase of fragments number

### Assembly of multiple fragments

After developing and optimizing the AFEAP cloning method, its efficiency and accuracy in assembling multiple fragments were evaluated. We tested the effects of DNA fragment number, final plasmid size, the molar ratio between longer and shorter DNA fragments, and transformation conditions.

**Fig. 3:**
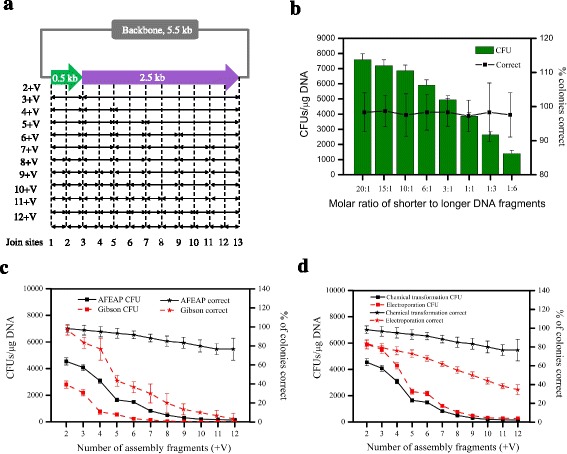
Assembly efficiency of multiple DNA fragments with AFEAP cloning. **a** Schematic details show the mechanism for the number of fragments characterization. The DNA sequence encoding T4 L and MCM6 proteins is split up into a number of fragments as shown by double-headed arrows. The number of fragments for each assembly is shown on the left of the double-headed arrows and the join sites for assembly is indicated on the below of dash lines. T4 L: T4 lysozyme. V: vector (backbone). **b** Effects of molar ratio between longer and shorter DNA fragments. **c** CFU and fidelity as a function of fragment number. **d** Effects of transformation conditions

To evaluate the effects of fragment number on efficiency, we built a pET22b-FLAG-T4 L-GGSGG_linker_-*MCM6* tandem construct, encoding T4 lysozyme (T4 L) [20] and MCM6 protein fused by a peptide linker, from varying number of DNA fragments (8.0 kb, Fig. 3a). PCR products were subjected to AFEAP cloning protocol as mentioned above (Fig. 1a). The join sites of each assembly were confirmed by DNA sequencing (Additional file 4: Figure S2). We first evaluated the effects of the molar ratio between the longer and shorter DNA fragments. It was shown that the molar ratio is critical for obtaining higher assembly efficiency. When increasing the molar ratio of shorter to longer DNA fragments from 1:6 to 20:1, the assembly efficiency increased 6-fold (Fig. 3b). But from the ratio of 10:1 onwards, the assembly efficiency increased slightly. In comparison, the assembly fidelity did not vary significantly for all conditions tested (Fig. 3b). As a result, the molar ratio was determined as 10:1 for high efficiency and low cost. And then we tested the effects of DNA fragment number. As we expected, the AFEAP assembly efficiency was shown a solid negative with increasing number of DNA fragments for assembly (Fig. 3c). The CFU per μg DNA dipped to around 100 when assembling 13 fragments (Fig. 3c). In contrast, the fidelity dropped slightly but remained >76% even for 13-fragment assembly. In comparison with commonly used Gibson method, AFEAP cloning method showed higher assembly efficiency (Fig. 3c), demonstrating the good performance of this new approach. Moreover, we evaluated the effects of transformation conditions on the assemble efficiency of multiple fragments with AFEAP method. Electroporation gave higher efficiencies, but lower fidelities which is similar as we mentioned above (Fig. 3d).

**Fig. 4:**
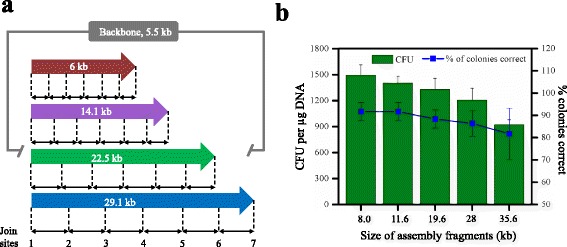
Effects of plasmid size on assembly efficiency. **a** The chosen 6 kb, 14.1 kb, 22.5 kb, and 29.1 kb gene clusters used for the size of construct characterization. **b** Efficiency as a function of construct size

Next, we evaluated the effects of the final plasmid size. We assembled four different plasmids of increasing sizes while keeping the number of fragments at six (Fig. 4a). The four chosen plasmids were an 11.5 kb plasmid pET22b harboring avermectin biosynthetic gene cluster (GenBank: AB032524.1) [21], a 19.6 kb pET22b harboring cosmomycin gene cluster (GenBank: DQ280500.1) [22], a 28 kb pET22b harboring the enterocin biosynthetic gene cluster (GenBank: AF254925.1) [23], and a 35.6 kb plasmid pET22b harboring the aureothin biosynthesis gene cluster *aurABCDEFGHI* (GenBank: AJ575648.1) [24]. The join sites of each assembly were confirmed by DNA sequencing (Additional file 5: Figure S3). As shown in Fig. 4b, the assembly efficiency showed a negative correlation with the size of plasmid assembled. When assembling plasmid size 8 kb, more than 1490 CFUs/μg ligated DNA were generated with an accuracy of 92%. In contrast, the assembly efficiency decreased to 1402, 1329, 1206, and 921 CFUs/μg when 11.5, 19.6, 28, and 35.6 kb plasmids were assembled, respectively. Even so, the accuracy is still more than 82% when assembling 35.6 kb plasmid, which confirms the high capacity of this sequence-independent assembly method.

### Construction of larger plasmid

**Fig. 5:**
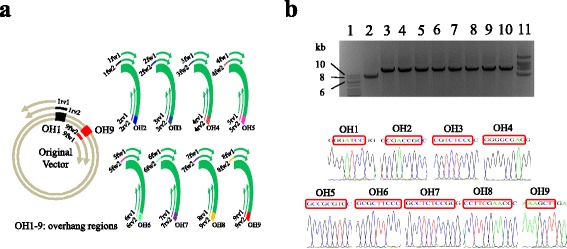
Construction of larger plasmid. **a** Schematic diagram of the assembly of 200 kb BAC with AFEAP cloning method. (**b**, upper panel) Agarose electrophoresis shows the PCR amplification using the primers as listed at Additional file 2: Table S2. Lane 1: 1 kb DNA ladder; Lane 2–9: PCR products from first-round PCRs; Lane 10: Annealing of PCR products from second-round PCRs before ligation; Lane 11: after ligation. DNA samples were electrophoresed in 1% agarose gel. (**b**, below panel) Sequencing validation of the re-joining junction sites. The overhang regions are marked by red boxes

As AFEAP cloning shows the ability for large fragment assembly, we tested the feasibility of AFEAP cloning to construct a bacterial artificial chromosome (BAC), which contains 200 kb DNA sequence insert. Accordingly, we proceeded to assemble the salinomycin biosynthesis cluster (200 kb; GenBank: HE586118.1) [25] from the *Streptomyces albus subsp. albus* (ATCC^®^ 55,161^™^) into pCC1BAC™ vector (Epicentre^®^) between the BamH I site (353–358) and the Hind III site (383–388) to form a BAC. As regular DNA polymerases only can amplify up to 40 kb with high fidelity, we plan to divide this 200 kb DNA sequence into 8 consecutive short ones, which are then assembled into BAC with AFEAP cloning. Figure 5a shows the strategy to construct BAC with AFEAP cloning method. Detailed cloning procedure can be found in Additional file 6: Supporting Information. Figure 5b shows the resulting DNA products by AFEAP cloning evaluated by 1% agarose gel electrophoresis, and the 8 consecutive DNA parts and the linear vector backbone were joined to one another and shifted to a higher molecular weight (Fig. 5b, lane 11). The presence of nine join sites was confirmed by DNA sequencing (Fig. 5b). 34 ± 13 CFUs/μg were obtained on transformation with an accuracy of 46.7 ± 4.7%. These results demonstrate that AFEAP cloning could be a powerful DNA assembly tool for multiple fragments, especially for large DNA up to 200 kb.

## Conclusions

We have developed an alternative DNA assembly method, named AFEAP cloning, which relies on two-round PCRs to insert complementary sticky ends to each 5′ end of DNA fragments for assembling multiple DNA fragments into functional parts. AFEAP cloning provides a powerful, efficient, seamless, and sequence-independent DNA assembly tool for multiple fragments up to 13 and large DNA up to 200 kb that expands synthetic biologist’s toolbox.

## Methods

### Bacterial strains, plasmids, and reagents

Host strain *E. coli* DH5α was obtained from Invitrogen (Carlsbad, CA, USA). The competent DH5α cells were prepared by using calcium chloride method [26]. Plasmid pET22b was purchased from Millipore Sigma (Billerica, MA, USA), and pCC1BAC™ vector was purchased from Epicentre^®^ (Madison, WI, USA). Genome DNAs were purchased from American Type Culture Collection (ATCC^®^, Manassas, VA, USA). Bacteria containing plasmids were cultured in Lysogeny Broth (LB; 10 g/L tryptone, 5 g/L yeast extract, 10 g/L NaCl) medium with appropriate antibiotics (kanamycin or ampicillin at 50 or 100 μg/ml) when necessary. Phusion^®^ high-fidelity DNA polymerase, DNA marker and T4 DNA ligase were purchased from New England Biolabs (NEB, Ipswich, MA, USA). G-HiFi^™^ DNA polymerase was purchased from SMOBIO Technology (Hsinchu, Taiwan). QIAquick PCR purification kit, QIAquick gel extraction kit, and QIAprep spin miniprep kit were purchased from Qiagen (Hilden, Germany). Gibson assembly master mix was from NEB (Ipswich, MA, USA).

### Primer design and DNA manipulation

All the primers used in this study were designed as shown in Fig. 1b, listed in Supplementary Additional file 2: Table S2, and synthesized by Shanghai Sangon Biotechnology. To determine the optimal length of overhang sequence for AFEAP cloning, the primers designed for first-round PCR flanking overhang region, and the primers designed for second-round PCR carry additional 0 to 20 nucleotides in their 5′ extension (See Fig. 2a for a schematic diagram of primer design). For multiple-fragment assembly, the primers designed for first-round PCR flank overhang regions, and for second-round PCR carry additional 5–8 nucleotides with 5′ end as G or C.

Unless otherwise stated, 50 μL PCR reactions were performed using Phusion^®^ high-fidelity DNA polymerase (NEB). The PCR conditions were listed in Additional files 7 and 8: Tables S3 and S4. The products of first-round PCR were purified by 1% agarose gel extraction with QIAquick gel extraction kit. The complementary DNA products from second-round PCRs were annealed without purification using the condition listed in Additional file 9: Table S5. The DNA fragments with complementary sticky ends were assembled via ligase cycling reaction, which was performed in a final volume of 20 μL using T4 DNA ligase following the standard protocol from New England Biolabs. In brief, the longer and shorter DNA fragments were mixed at a molar ratio of 1:10. The mixture was incubated at room temperature for 2 h. After heat inactivation at 65 °C for 10 min, the reaction was chilled on ice.

### Plasmid transformation, isolation, and sequence

After ligation, 10 μL of the ligation products was directly added to 100 μL of competent DH5α cells, incubated for 15 min on ice, heat-shocked at 42 °C for 1 min and then transferred to ice for 5 min. After adding 500 μL of LB medium the cells were subsequently incubated at 37 °C and 200 rpm for 1 h. After incubation, cells were pelleted. The supernatant was removed leaving 100 μl and the pellet was resuspended in the remaining supernatant that was then spread onto a LB agar plate containing ampicillin (100 μg/ml) or kanamycin (50 μg/ml). After incubating the plates overnight at 37 °C, for each transformation we selected ten colonies at random and the plasmids were isolated with QIAprep spin miniprep kit. For each assembly the re-joining junction sites were validated by DNA sequence to ensure accuracy of corrected assembly.

### Gibson assembly

Gibson assembly was carried out according to the manufacturer’s protocol (Gibson Assembly Master Mix Instruction Manual, NEB). In brief, the longer and shorter DNA fragments were mixed at a molar ratio of 1:3–1:10. DNA assembly was performed in a total volume of 20 μL containing 1 pmol DNA fragments and 10 μL Gibson assembly master mix. Samples were incubated in a thermocycler at 50 °C for 60 min. Following incubation, samples were stored on ice or at −20 °C for subsequent transformation.

### Electroporation

Electroporation was carried out followed a protocol from New England Biolabs (NEB, Ipswich, MA, USA). In brief, the ligation mixture was purified with QIAquick PCR purification kit, and transformed into electrocompetent DH5α cells by using a Gene Pulsar apparatus (Bio-Rad).

## Additional files

Additional file 1: Table S1. Comparisons of AFEAP cloning with common DNA assembly methods. (DOCX 23 kb)

Additional file 2: Table S2. Primers used in this work. (DOCX 32 kb)

Additional file 3: Figure S1. Sequencing validation of assemble with various overhangs. (a)-(g) various overhang sizes; (h) overhang designed as 5′ end of G/C; (i) overhang designed as 5′ end of A/T. Overhang regions were marked by red dashed line rectangles. (DOCX 2777 kb)

Additional file 4: Figure S2. Sequencing validation of number of fragments characterization. The join sites were shown as S1 to S13, and number of fragments for assembly was shown as 2 + V to 12 + V. The overhang sequences were shown. V: vector backbone. (DOCX 11884 kb)

Additional file 5: Figure S3. Sequencing validation of plasmid sizes characterization. Five join sites are S1, S2, S3, S4, and S5. (a) 11.5 kb plasmid; (b) 19.6 kb plasmid; (c) 28 kb plasmid; (d) 34.6 kb plasmid. The overhang sequences were shown. (DOCX 3815 kb)

Additional file 6: Supporting Information. (DOCX 32 kb)

Additional file 7: Table S3. PCR conditions. (DOCX 14 kb)

Additional file 8: Table S4. PCR thermocycling conditions. (DOCX 13 kb)

Additional file 9: Table S5. PCR product reannealing conditions. (DOCX 14 kb)
