# Supplementary material for: AFEAP cloning: a precise and efficient method for large DNA sequence assembly
Source: BMC Biotechnol. 2017 Nov 14;17:81. doi: 10.1186/s12896-017-0394-x (PMC5686892; doi:10.1186/s12896-017-0394-x)
Supplement: Supplementary file 1 — Comparisons of AFEAP cloning with common DNA assembly methods. (DOCX 23 kb) [file 12896_2017_394_MOESM1_ESM.docx]

**Table S1** Comparisons of AFEAP cloning with common DNA assembly methods

|  | Capability^a^ | Scarless | Step(s) | Reference |
| --- | --- | --- | --- | --- |
| DATEL | 2−10 DNA fragments with fidelity between 74 and 100% | yes | 1 | [^2^](#_ENREF_2) |
| CPEC | 9 kb plasmid from 5 fragments at ∼90% fidelity | yes | 1 | [^3^](#_ENREF_3) |
| SLIC | 8 kb plasmid from 10 fragments at ∼20% fidelity | yes | 2 | [^4^](#_ENREF_4) |
| Gibson | Up to several hundred kilobases, but did not enable assembly of more than four DNA parts with more than 50% of clones being correct. | yes | 1 | [^5^](#_ENREF_5) |
| USER | 8 kb plasmid from 11 fragments at ∼60% fidelity | yes | 1 | [^6^](#_ENREF_6) |
| Golden | At least nine separate DNA fragments together into an acceptor vector, with 90% of recombinant clones obtained containing the desired construct. | yes | 1 | [^7^](#_ENREF_7) |
| LCR | up to 12 DNA parts with 60–100% of individual clones being correct | yes | 1 | [^8^](#_ENREF_8) |
| DNA assembler | ∼9 kb DNA consisting of three genes, ∼11 kb DNA consisting of five genes, and ∼19 kb consisting of eight genes with high efficiencies (70–100%) | yes | 3 | [^9^](#_ENREF_9) |
| OEPR | Large DNA fragments up to 6 kb or multiple DNA fragments up to two 3 kb, three 2 kb and four 1 kb into vectors (8 kb tested). | yes | 1 | [^10^](#_ENREF_10) |
| TPA | 7 kb plasmid from 10 fragments at ∼80% fidelity and 31 kb plasmid from five fragments at ∼50% fidelity. | yes | 1 | [^11^](#_ENREF_11) |
| AFEAP | 8 kb plasmid from 13 fragments at ∼80% fidelity and 35.6 kb plasmid from six fragments at ∼82% fidelity, 200 kb plasmid from 9 fragments at ∼47%. | yes | 1 | This study |

^a^ The largest demonstrated number of fragments, plasmids size, and fidelity are reported in references.
