# Supplementary material for: AFEAP cloning: a precise and efficient method for large DNA sequence assembly
Source: BMC Biotechnol. 2017 Nov 14;17:81. doi: 10.1186/s12896-017-0394-x (PMC5686892; doi:10.1186/s12896-017-0394-x)
Supplement: Supplementary file 2 — Primers used in this work. (DOCX 32 kb) [file 12896_2017_394_MOESM2_ESM.docx]

**Table S2** Primers used in this work

| Name | Primers (5'→3')* |
| --- | --- |
| **Various length of overhangs** | |
| OHtestfw1 | TATTCTGCAGATATCCAGCACAGTGG |
| OHtestrv1 | CTCAATCTTCGAGCAAGTAGTTAG |
| OHtest2fw2 | **AA**TATTCTGCAGATATCCAGCACAGTGG |
| OHtest2rv2 | **TT**CTCAATCTTCGAGCAAGTAGTTAG |
| OHtest3fw2 | **GAA**TATTCTGCAGATATCCAGCACAGTGG |
| OHtest3rv2 | **TTC**CTCAATCTTCGAGCAAGTAGTTAG |
| OHtest4fw2 | **AGAA**TATTCTGCAGATATCCAGCACAGTGG |
| OHtest4rv2 | **TTCT**CTCAATCTTCGAGCAAGTAGTTAG |
| OHtest5fw2 | **GAGAA**TATTCTGCAGATATCCAGCACAGTGG |
| OHtest5rv2 | **TTCTC**CTCAATCTTCGAGCAAGTAGTTAG |
| OHtest8fw2 | **ATTGAGAA**TATTCTGCAGATATCCAGCACAGTGG |
| OHtest8rv2 | **TTCTCAAT**CTCAATCTTCGAGCAAGTAGTTAG |
| OHtest10fw2 | **AGATTGAGAA**TATTCTGCAGATATCCAGCACAGTGG |
| OHtest10rv2 | **TTCTCAATCT**CTCAATCTTCGAGCAAGTAGTTAG |
| OHtest14fw2 | **TCGAAGATTGAGAA**TATTCTGCAGATATCCAGCACAGTGG |
| OHtest14rv2 | **TTCTCAATCTTCGA**CTCAATCTTCGAGCAAGTAGTTAG |
| OHtest20fw2 | **CATTGCTCGAAGATTGAGAA**TATTCTGCAGATATCCAGCACAGTGG |
| OHtest20rv2 | **TTCTCAATCTTCGAGCAATG**CTCAATCTTCGAGCAAGTAGTTAG |
| **Analysis of the effect of 5’ end of the overhang** | |
| OHtestGCfw2 | **GTTGAGAC**TATTCTGCAGATATCCAGCACAGTGG |
| OHtestGCrv2 | **CTCTCAAG**CTCAATCTTCGAGCAAGTAGTTAG |
| OHtestATfw2 | **ATTGAGAT**TATTCTGCAGATATCCAGCACAGTGG |
| OHtestATrv2 | **ATCTCAAT**CTCAATCTTCGAGCAAGTAGTTAG |
| **Assembly of 8 kb plasmid** | |
| Primers for the assembly of site 1 | |
| 8Site1fw1 | GACTACAAGGATGAAGAGGACAAGAACATCTTTGAAATGCTGCGTATTG |
| 8Site1rv1 | TATATCTCCTTCTTAAAGTTAAAC |
| 8Site1fw2 | **CATATG**GACTACAAGGATGAAGAGGACAAGAAC |
| 8Site1rv2 | **CATATG**TATATCTCCTTCTTAAAGTTAAAC |
| Primers for the assembly of site 2 | |
| 8Site2fw1 | GTGGTATTCTGCGCAATGCAAAAC |
| 8Site2rv1 | TGCGTCCACATCCTGGTTAAAC |
| 8Site2fw2 | **GCTGTTC**TGCGTCCACATCCTGGTTAAAC |
| 8Site2rv2 | **GAACAGC**CACCACCACCACCACCACTGAGATC |
| Primers for the assembly of site 3 | |
| 8Site3fw1 | GGCGGATCAGGCGGTGACCTCGCGGCGGCAGCGGAGCC |
| 8Site3rv1 | GTCCCAGGTGCCGGTGCGAAAGGTAATTG |
| 8Site3fw2 | **GCCTAT**GGCGGATCAGGCGGTGACCTCG |
| 8Site3rv2 | **ATAGGC**GTCCCAGGTGCCGGTGCGAAAGGTAATTG |
| Primers for the assembly of site 4 | |
| 8Site4fw1 | GAGTTCTATAGAGTTTACCCTTAC |
| 8Site4rv1 | AATGGTGGTGGAAAGTTGCTGG |
| 8Site4fw2 | **CAAGAG**GAGTTCTATAGAGTTTACCCTTAC |
| 8Site4rv2 | **CTCTTG**AATGGTGGTGGAAAGTTGCTGG |
| Primers for the assembly of site 5 | |
| 8Site5fw1 | GATGTAGAACAGCAGTTCAAATAC |
| 8Site5rv1 | ACTGTCTGACAGTCCAAGCAC |
| 8Site5fw2 | **GATCAGG**GATGTAGAACAGCAGTTCAAATAC |
| 8Site5rv2 | **CCTGATC**ACTGTCTGACAGTCCAAGCAC |
| Primers for the assembly of site 6 | |
| 8Site6fw1 | CCTGACGTCTCCAAGCTTAGCAC |
| 8Site6rv1 | AATCAGTGTCCCTGTAAAGTCAC |
| 8Site6fw2 | **GTTGTG**CCTGACGTCTCCAAGCTTAGCAC |
| 8Site6rv2 | **CACAAC**AATCAGTGTCCCTGTAAAGTCAC |
| Primers for the assembly of site 7 | |
| 8Site7fw1 | GAGAAAGTGTTTGAGATGAGTC |
| 8Site7rv1 | TTTCACAGTCATTTGGTTCTTAATG |
| 8Site7fw2 | **GAATGG**GAGAAAGTGTTTGAGATGAGTC |
| 8Site7rv2 | **CCATTC**TTTCACAGTCATTTGGTTCTTAATG |
| Primers for the assembly of site 8 | |
| 8Site8fw1 | CCAGAGCTGTCTACACCAGTGG |
| 8Site8rv1 | AACTCCTCCACGTGCTTGAGAAATTG |
| 8Site8fw2 | **CAGCC**CCAGAGCTGTCTACACCAGTGG |
| 8Site8rv2 | **GGCTG**AACTCCTCCACGTGCTTGAGAAATTG |
| Primers for the assembly of site 9 | |
| 8Site9fw1 | CATTTTGGCAGCAGCAAACCCAATC |
| 8Site9rv1 | CGGGCGTTCAGAGTAGCCTTCAC |
| 8Site9fw2 | **GACGTC**CATTTTGGCAGCAGCAAACCCAATC |
| 8Site9rv2 | **GACGTC**CGGGCGTTCAGAGTAGCCTTCAC |
| Primers for the assembly of site 10 | |
| 8Site10fw1 | CAAGACAGTTTAAACCCAAGATTTC |
| 8Site10rv1 | AGAAGATATCTTCTGATATCATC |
| 8Site10fw2 | **CTTTG**CAAGACAGTTTAAACCCAAGATTTC |
| 8Site10rv2 | **CAAAG**AGAAGATATCTTCTGATATCATC |
| Primers for the assembly of site 11 | |
| 8Site11fw1 | GTGTGGAAACACCTGATGTCAATC |
| 8Site11rv1 | GATTGACATCAGGTGTTTCCACAC |
| 8Site11fw2 | **CATCC**GTGTGGAAACACCTGATGTCAATC |
| 8Site11rv2 | **GGATG**GATTGACATCAGGTGTTTCCACAC |
| Primers for the assembly of site 12 | |
| 8Site12fw1 | GAGTCAGCATTAAAGAGGAGCG |
| 8Site12rv1 | TTCTTCTTCCACCTTTCTGAGG |
| 8Site12fw2 | **GAGGAC**GAGTCAGCATTAAAGAGGAGCG |
| 8Site12rv2 | **GTCCTC**TTCTTCTTCCACCTTTCTGAGG |
| Primers for the assembly of site 13 | |
| 8Site13fw1 | CACCACCACCACCACCACTGAGATC |
| 8Site13rv1 | ATCTTCGAGCAAGTAGTTAGGG |
| 8Site13fw2 | **CTCGAG**CACCACCACCACCACCACTGAGATC |
| 8Site13rv2 | **CTCGAG**ATCTTCGAGCAAGTAGTTAGGG |
| **Assembly of 11.5 kb plasmid** | |
| Primers for the assembly of site 1 | |
| 11.5Site1fw1 | ACCGGTCACCCGGTATTCCATTC |
| 11.5Site1rv1 | TATATCTCCTTCTTAAAGTTAAAC |
| 11.5Site1fw2 | **CATATG**ACCGGTCACCCGGTATTCCATTC |
| 11.5Site1rv2 | **CATATG**TATATCTCCTTCTTAAAGTTAAAC |
| Primers for the assembly of site 2 | |
| 11.5Site2fw1 | CTGCTGCGGGCCCTGCTCGAAG |
| 11.5Site2rv1 | GTTGCCCCCGGTGACGGTCAG |
| 11.5Site2fw2 | **CCGCTG**CTGCTGCGGGCCCTGCTCGAAG |
| 11.5Site2rv2 | **CAGCGG**GTTGCCCCCGGTGACGGTCAG |
| Primers for the assembly of site 3 | |
| 11.5Site3fw1 | CCGGAAACGGCACCAGCGAGGC |
| 11.5Site3rv1 | CCCACAAGGCCGCTGTCGGCTG |
| 11.5Site3fw2 | **CCCTGC**CCGGAAACGGCACCAGCGAGGC |
| 11.5Site3rv2 | **GCAGGG**CCCACAAGGCCGCTGTCGGCTG |
| Primers for the assembly of site 4 | |
| 11.5Site4fw1 | CGGGCGGCAACACCAACCGTGAG |
| 11.5Site4rv1 | TGTCGCGACTCGCATCTCGGACTC |
| 11.5Site4fw2 | **CTGGCGG**CGGGCGGCAACACCAACCGTGAG |
| 11.5Site4rv2 | **CCGCCAG**TGTCGCGACTCGCATCTCGGACTC |
| Primers for the assembly of site 5 | |
| 11.5Site5fw1 | GGAAAGCAGGCACGGTGTTCC |
| 11.5Site5rv1 | GGAATTTCATCGTGGCGGATC |
| 11.5Site5fw2 | **CCTTTTC**GGAAAGCAGGCACGGTGTTCC |
| 11.5Site5rv2 | **GAAAAGG**GGAATTTCATCGTGGCGGATC |
| Primers for the assembly of site 6 | |
| 11.5Site6fw1 | CGCGTACTCGTCGAGGTGCTCGTTC |
| 11.5Site6rv1 | TTCGGGATCGGCGTCGCGGAG |
| 11.5Site6fw2 | **GGCCGC**CGCGTACTCGTCGAGGTGCTCGTTC |
| 11.5Site6rv2 | **GCGGCC**TTCGGGATCGGCGTCGCGGAG |
| Primers for the assembly of site 7 | |
| 11.5Site7fw1 | CACCACCACCACCACCACTGAGATC |
| 11.5Site7rv1 | CTGACGGCCGGGCATCAATGTC |
| 11.5Site7fw2 | **CTCGAG**CACCACCACCACCACCACTGAGATC |
| 11.5Site7rv2 | **CTCGAG**CTGACGGCCGGGCATCAATGTC |
| **Assembly of 19.6 kb plasmid** | |
| Primers for the assembly of site 1 | |
| 19.6Site1fw1 | GACCCCAGCTGGACGACCCGCAG |
| 19.6Site1rv1 | TATATCTCCTTCTTAAAGTTAAAC |
| 19.6Site1fw2 | **CATATG**GACCCCAGCTGGACGACCCGCAG |
| 19.6Site1rv2 | **CATATG**TATATCTCCTTCTTAAAGTTAAAC |
| Primers for the assembly of site 2 | |
| 19.6Site2fw1 | GGCGAACGACCCGTATGCGATG |
| 19.6Site2rv1 | GGGTTGGGATGCGGGTACTCTTC |
| 19.6Site2fw2 | **CTGTTCC**GGCGAACGACCCGTATGCGATG |
| 19.6Site2rv2 | **GGAACAG**GGGTTGGGATGCGGGTACTCTTC |
| Primers for the assembly of site 3 | |
| 19.6Site3fw1 | CATCGGGGTGGTGGCCCCCGG |
| 19.6Site3rv1 | ATCACGACCCGCCGGGTCATC |
| 19.6Site3fw2 | **CACCGG**CATCGGGGTGGTGGCCCCCGG |
| 19.6Site3rv2 | **CCGGTG**ATCACGACCCGCCGGGTCATC |
| Primers for the assembly of site 4 | |
| 19.6Site4fw1 | TCCGGCACGGCGGCCCTGGTCC |
| 19.6Site4rv1 | CCGGGGCCGTCCGAGGACGAGG |
| 19.6Site4fw2 | **CCGGTC**TCCGGCACGGCGGCCCTGGTCC |
| 19.6Site4rv2 | **GACCGG**CCGGGGCCGTCCGAGGACGAGG |
| Primers for the assembly of site 5 | |
| 19.6Site5fw1 | CGATCAGACCAACAGCGAGTTGG |
| 19.6Site5rv1 | ATGACACCGTCCTTGGGAGAAG |
| 19.6Site5fw2 | **GATCAGG**CGATCAGACCAACAGCGAGTTGG |
| 19.6Site5rv2 | **CCTGATC**ATGACACCGTCCTTGGGAGAAG |
| Primers for the assembly of site 6 | |
| 19.6Site6fw1 | GGCGGGGCTGCACCTGCACTCCG |
| 19.6Site6rv1 | TCCTCCACCCGCTGCGCGCGG |
| 19.6Site6fw2 | **GCTGGG**GGCGGGGCTGCACCTGCACTCCG |
| 19.6Site6rv2 | **CCCAGC**TCCTCCACCCGCTGCGCGCGG |
| Primers for the assembly of site 7 | |
| 19.6Site7fw1 | CACCACCACCACCACCACTGAGATC |
| 19.6Site7rv1 | GCGGTCATGACTGGGCGACCTC |
| 19.6Site7fw2 | **CTCGAG**CACCACCACCACCACCACTGAGATC |
| 19.6Site7rv2 | **CTCGAG**GCGGTCATGACTGGGCGACCTC |
| **Assembly of 28 kb plasmid** | |
| Primers for the assembly of site 1 | |
| 28Site1fw1 | GCCGACACTCCCGCCTCGGACAAACG |
| 28Site1rv1 | TATATCTCCTTCTTAAAGTTAAAC |
| 28Site1fw2 | **CATATG**GCCGACACTCCCGCCTCGGACAAACG |
| 28Site1rv2 | **CATATG**TATATCTCCTTCTTAAAGTTAAAC |
| Primers for the assembly of site 2 | |
| 28Site2fw1 | GTGAGCGGCAGCATCGGGCACCCCTG |
| 28Site2rv1 | GGCCAAAGGCACGCAGCCTGGGG |
| 28Site2fw2 | **CGTGAC**GTGAGCGGCAGCATCGGGCACCCCTG |
| 28Site2rv2 | **GTCACG**GGCCAAAGGCACGCAGCCTGGG |
| Primers for the assembly of site 3 | |
| 28Site3fw1 | CGGTGCGCGGCGAACTTGAGC |
| 28Site3rv1 | CCGCTACACCCCGCGCTGCGTGC |
| 28Site3fw2 | **CGCAGG**CGGTGCGCGGCGAACTTGAGC |
| 28Site3rv2 | **CCTGCG**CCGCTACACCCCGCGCTGCGTGC |
| Primers for the assembly of site 4 | |
| 28Site4fw1 | GTCCTCGCGCGCGGCGCCGGCGGC |
| 28Site4rv1 | CTCGACAGGGGGCACGTGCGTG |
| 28Site4fw2 | **GGCCCTC**GTCCTCGCGCGCGGCGCCGGCGGC |
| 28Site4rv2 | **GAGGGCC**CTCGACAGGGGGCACGTGCGTG |
| Primers for the assembly of site 5 | |
| 28Site5fw1 | CTGGAGACCATCGAACGAGTACG |
| 28Site5rv1 | TGCGGTGCCGGAAACCCGGG |
| 28Site5fw2 | **GATCAGG**CTGGAGACCATCGAACGAGTACG |
| 28Site5rv2 | **CCTGATC**TGCGGTGCCGGAAACCCGGG |
| Primers for the assembly of site 6 | |
| 28Site6fw1 | GAAACTGCCCGAGCCCAGCGGG |
| 28Site6rv1 | AGGGTCGCCACCGAGCCGGTGG |
| 28Site6fw2 | **GGCCCG**GAAACTGCCCGAGCCCAGCGGG |
| 28Site6rv2 | **CGGGCC**AGGGTCGCCACCGAGCCGGTGG |
| Primers for the assembly of site 7 | |
| 28Site7fw1 | CACCACCACCACCACCACTGAGATC |
| 28Site7rv1 | GGAATTTCAATGATCCTTGG |
| 28Site7fw2 | **CTCGAG**CACCACCACCACCACCACTGAGATC |
| 28Site7rv2 | **CTCGAG**GGAATTTCAATGATCCTTGG |
| **Assembly of 35.6 kb plasmid** | |
| Primers for the assembly of site 1 | |
| 35.6Site1fw1 | GATCAGCTCGTCCCGTTCGGAG |
| 35.6Site1rv1 | TATATCTCCTTCTTAAAGTTAAAC |
| 35.6Site1fw2 | **CATATG**GATCAGCTCGTCCCGTTCGGAG |
| 35.6Site1rv2 | **CATATG**TATATCTCCTTCTTAAAGTTAAAC |
| Primers for the assembly of site 2 | |
| 35.6Site2fw1 | CCGCTGGAGATCGTGCCGTTCG |
| 35.6Site2rv1 | GGGGACGAGCGTGTCGTAGTCG |
| 35.6Site2fw2 | **GACTAC**CCGCTGGAGATCGTGCCGTTCG |
| 35.6Site2rv2 | **GTAGTC**GGGGACGAGCGTGTCGTAGTCG |
| Primers for the assembly of site 3 | |
| 35.6Site3fw1 | CCGGCCGCCGGTGACGCCGCTG |
| 35.6Site3rv1 | GCGAGGACGACGACGTCGGGCGCCGTG |
| 35.6Site3fw2 | **GGAGCTG**CCGGCCGCCGGTGACGCCGCTG |
| 35.6Site3rv2 | **CAGCTCC**GCGAGGACGACGACGTCGGGCGCCGTG |
| Primers for the assembly of site 4 | |
| 35.6Site4fw1 | GGCGTCGCCGGGCCGGACGCCC |
| 35.6Site4rv1 | TGCCGCCAGGTGATCTCGCCGG |
| 35.6Site4fw2 | **CGAGCTC**GGCGTCGCCGGGCCGGACGCCC |
| 35.6Site4rv2 | **GAGCTCG**TGCCGCCAGGTGATCTCGCCGG |
| Primers for the assembly of site 5 | |
| 35.6Site5fw1 | CCGGTTCATCGAGATGGGCAAG |
| 35.6Site5rv1 | GGCCGCACCAGGCGCAGCGAG |
| 35.6Site5fw2 | **GGGCGG**CCGGTTCATCGAGATGGGCAAG |
| 35.6Site5rv2 | **CCGCCC**GGCCGCACCAGGCGCAGCGAG |
| Primers for the assembly of site 6 | |
| 35.6Site6fw1 | CAGTTCGACCCGCTCCTCTTC |
| 35.6Site6rv1 | GTCGCGCAGGAAGCCGCCGCGGTTG |
| 35.6Site6fw2 | **GCCGAC**CAGTTCGACCCGCTCCTCTTC |
| 35.6Site6rv2 | **GTCGGC**GTCGCGCAGGAAGCCGCCGCGGTTG |
| Primers for the assembly of site 7 | |
| 35.6Site7fw1 | CACCACCACCACCACCACTGAGATC |
| 35.6Site7rv1 | TCAGTCAGTCGTCCAGGCGCGCCAG |
| 35.6Site7fw2 | **CTCGAG**CACCACCACCACCACCACTGAGATC |
| 35.6Site7rv2 | **CTCGAG**TCAGTCAGTCGTCCAGGCGCGCCAG |
| **200 kb BAC assembly** | |
| Primers for the assembly of site 1 | |
| BACSite1fw1 | GGATGCGAAGGACGCGCTGCGCAAGG |
| BACSite1rv1 | CCGGGTACCGAGCTCGAATTCGC |
| BACSite1fw2 | **GGATCC**GGATGCGAAGGACGCGCTGCGCAAGG |
| BACSite1rv2 | **GGATCC**CCGGGTACCGAGCTCGAATTCGC |
| Primers for the assembly of site 2 | |
| BACSite2fw1 | CGGTACGCCCCCGTGGTGATCACCG |
| BACSite2rv1 | AGACGGCCTGCCCGGAGCCGGCGAG |
| BACSite2fw2 | **GCGACCGC**CGGTACGCCCCCGTGGTGATCACCG |
| BACSite2rv2 | **GCGGTCGC**AGACGGCCTGCCCGGAGCCGGCGAG |
| Primers for the assembly of site 3 | |
| BACSite3fw1 | CCGCCGAACGCGGCCACGAGGTCAC |
| BACSite3rv1 | GCGCAGGCGAGCCCGGCGGGAC |
| BACSite3fw2 | **CGTCTCCG**CCGCCGAACGCGGCCACGAGGTCAC |
| BACSite3rv2 | **CGGAGACG**GCGCAGGCGAGCCCGGCGGGAC |
| Primers for the assembly of site 4 | |
| BACSite4fw1 | GAGCTCGCTGCCTTGACCCCCCG |
| BACSite4rv1 | GCCGGGCTCAGCCGCTGCCGAG |
| BACSite4fw2 | **GGGGCGAG**GAGCTCGCTGCCTTGACCCCCCG |
| BACSite4rv2 | **CTCGCCCC**GCCGGGCTCAGCCGCTGCCGAG |
| Primers for the assembly of site 5 | |
| BACSite5fw1 | CAGGCTGCGCTGGATCTGCAGCGAG |
| BACSite5rv1 | CTTCCACGAGGAGTCGCTCGCCC |
| BACSite5fw2 | **GCCGCGTG**CAGGCTGCGCTGGATCTGCAGCGAG |
| BACSite5rv2 | **CACGCGGC**CTTCCACGAGGAGTCGCTCGCCC |
| Primers for the assembly of site 6 | |
| BACSite6fw1 | GCGGGTGGCGCGATGGTCTCCATAC |
| BACSite6rv1 | CTCCATCAACCGACCACGAGCAG |
| BACSite6fw2 | **GCGCTTCCC**GCGGGTGGCGCGATGGTCTCCATAC |
| BACSite6rv2 | **GGGAAGCGC**CTCCATCAACCGACCACGAGCAG |
| Primers for the assembly of site 7 | |
| BACSite7fw1 | GCTCGACGTGGACGCGGTCGAGGCC |
| BACSite7rv1 | GGGCGTTGGCGAGGGCCTGGCGGATC |
| BACSite7fw2 | **GCCTCTCCGC**GCTCGACGTGGACGCGGTCGAGGCC |
| BACSite7rv2 | **GCGGAGAGGC**GGGCGTTGGCGAGGGCCTGGCGGATC |
| Primers for the assembly of site 8 | |
| BACSite8fw1 | CGCCGGTATCGACCCGGGCACCCTCAAG |
| BACSite8rv1 | TCTCCCAGGCCGATTCGAGCAGCAGC |
| BACSite8fw2 | **CCTTCGAACG**CGCCGGTATCGACCCGGGCACCCTCAAG |
| BACSite8rv2 | **CGTTCGAAGG**TCTCCCAGGCCGATTCGAGCAGCAGC |
| Primers for the assembly of site 9 | |
| BACSite9fw1 | GAGTATTCTATAGTCTCACCTAAATAG |
| BACSite9rv1 | TTGCGTGCCGCCGCCGCGGCGC |
| BACSite9fw2 | **AAGCTT**GAGTATTCTATAGTCTCACCTAAATAG |
| BACSite9rv2 | **AAGCTT**TTGCGTGCCGCCGCCGCGGCGC |

^*^ Bold purple and green letters show overhang sequences.
