# Supplementary material for: AFEAP cloning: a precise and efficient method for large DNA sequence assembly
Source: BMC Biotechnol. 2017 Nov 14;17:81. doi: 10.1186/s12896-017-0394-x (PMC5686892; doi:10.1186/s12896-017-0394-x)
Supplement: Supplementary file 3 — Sequencing validation of assemble with various overhangs. (a)-(g) various overhang sizes; (h) overhang designed as 5′ end of G/C; (i) overhang designed as 5′ end of A/T. Overhang regions were marked by red dashed line rectangles. (DOCX 2777 kb) [file 12896_2017_394_MOESM3_ESM.docx]

**Figure S1.** Sequencing validation of assemble with various overhangs. (a)-(g) various overhang sizes; (h) overhang designed as 5' end of G/C; (i) overhang designed as 5' end of A/T. Overhang regions were marked by red dashed line rectangles.
