# Supplementary material for: AFEAP cloning: a precise and efficient method for large DNA sequence assembly
Source: BMC Biotechnol. 2017 Nov 14;17:81. doi: 10.1186/s12896-017-0394-x (PMC5686892; doi:10.1186/s12896-017-0394-x)
Supplement: Supplementary file 4 — Sequencing validation of number of fragments characterization. The join sites were shown as S1 to S13, and number of fragments for assembly was shown as 2 + V to 12 + V. The overhang sequences were shown. V: vector backbone. (DOCX 11884 kb) [file 12896_2017_394_MOESM4_ESM.docx]

**Figure S2.** Sequencing validation of number of fragments characterization. The join sites were shown as S1 to S13, and number of fragments for assembly was shown as 2+V to 12+V. The overhang sequences were shown. V: vector backbone.
