# Supplementary material for: AFEAP cloning: a precise and efficient method for large DNA sequence assembly
Source: BMC Biotechnol. 2017 Nov 14;17:81. doi: 10.1186/s12896-017-0394-x (PMC5686892; doi:10.1186/s12896-017-0394-x)
Supplement: Supplementary file 5 — Sequencing validation of plasmid sizes characterization. Five join sites are S1, S2, S3, S4, and S5. (a) 11.5 kb plasmid; (b) 19.6 kb plasmid; (c) 28 kb plasmid; (d) 34.6 kb plasmid. The overhang sequences were shown. (DOCX 3815 kb) [file 12896_2017_394_MOESM5_ESM.docx]

**Figure S3.** Sequencing validation of plasmid sizes characterization. Five join sites are S1, S2, S3, S4, and S5. (a) 11.5 kb plasmid; (b) 19.6 kb plasmid; (c) 28 kb plasmid; (d) 34.6 kb plasmid. The overhang sequences were shown.
