# Supplementary material for: AFEAP cloning: a precise and efficient method for large DNA sequence assembly
Source: BMC Biotechnol. 2017 Nov 14;17:81. doi: 10.1186/s12896-017-0394-x (PMC5686892; doi:10.1186/s12896-017-0394-x)
Supplement: Supplementary file 6 — Supporting Information. (DOCX 32 kb) [file 12896_2017_394_MOESM6_ESM.docx]

**Additional file methods**

**Optimization of key parameters for AFEAP cloning.** For the experiment of optimizing overhang sizes for efficient assembly, we designed a set of special linear DNAs with varying size from 5.5 to 30 kb (total five different DNAs), but each containing same sequences at 5' (5'CTAACTACTTGCTCGAAGATTGAG3') and 3' (5'TTCTGCAGATATCCAGCACAGTGG3') terminal for convenient primer designing (Figure 1c). Primers were designed to add 0 to 20 bp (total nine different overhangs) overhang adapter sequence at 5' ends of DNA molecules. For each test, we performed AFEAP cloning procedures as shown at Figure 1a. PCR conditions were listed in Supplementary Table S3, and thermocycling conditions were listed in the Supplementary Table S4. According, step1: five PCRs were performed to generate five double-stranded DNA fragments used primer pairs OHtestfw1/OHtestrv1 and 5.5 kb, 8.0 kb, 15 kb, 20 kb, or 30 kb DNA as template. PCR products were gel purified. Step 2: Two single-primer PCRs in parallel were performed to generate two complementary single-stranded DNA fragments using each purified fragment generated in the Step 1 as template and single primer of OHtest1fw2 or OHtest1rv2, OHtest2fw2 or OHtest2rv2, OHtest3fw2 or OHtest3rv2, OHtest4fw2 or OHtest4rv2, OHtest5fw2 or OHtest5rv2, OHtest8fw2 or OHtest8rv2, OHtest10fw2 or OHtest10rv2, OHtest14fw2 or OHtest14rv2, or OHtest20fw2 or OHtest20rv2 (for detailed primer sequences see Supplementary Table S2). Step 3: The newly synthesized complementary PCR products in step 2 were annealed using the conditions as shown in Supplementary Table S5. Step 4: total 1 µg of annealed DNAs with nick were sealed by T4 DNA ligase (NEB) to form transformable plasmid used protocol from NEB. Reconstituted vector was transformed into competent *E.coli* cells. The colonies forming were counted, and the join sites were confirmed by DNA sequence (Supplementary Figure S1a-g).

To determine the effect of the 5' end of the overhang as G/C or A/T on the efficiency of assembly with AFEAP method, we designed four primers: OHtestGCfw2, OHtestGCrv2, OHtestATfw2, and OHtestATrv2 (See Supplementary Table S2). We ran AFEAP cloning as shown at Figure 1a. PCR conditions were listed in Supplementary Table S3, and thermocycling conditions were listed in the Supplementary Table S4. According, step1: 5 PCRs were performed to generate five double-stranded DNA fragments used primer pairs: OHtestfw1 and OHtestrv1, and 5.5 kb, 8.0 kb, 15 kb, 20 kb, or 30 kb DNA fragments as templates. PCR products were gel purified. Step 2: Two single-primer PCRs in parallel were performed to generate two complementary single-stranded DNA fragments using each purified fragment generated in the Step 1 as template and single primer of OHtestGCfw2 or OHtestGCrv2, or OHtestATfw2 or OHtestATrv2. Step 3: The newly synthesized complementary single-stranded DNA fragments were annealed using the conditions as shown in Supplementary Table S5. Step 4: total 1 µg of annealed DNAs with nick were sealed by T4 DNA ligase (NEB) to form transformable plasmid used protocol from NEB. Reconstituted vector was transformed into competent *E.coli* cells. The colonies were counted, and the join sites were confirmed by DNA sequencing (Supplementary Figure S1h and i).

To determine the effect of ligation on the assembly, we ran AFEAP cloning as shown at Figure 1a. According, step1: Five PCRs were performed to generate five double-stranded DNA fragments used primer pairs: OHtestfw1 and OHtestrv1, and 5.5 kb, 8.0 kb, 15 kb, 20 kb, or 30 kb DNA fragments as templates. PCR products were gel purified. Step 2: Two single-primer PCRs in parallel were performed to generate two complementary single-stranded DNA fragments using each purified fragment generated in the Step 1 as template and single primer of OHtest5fw2 or OHtest5rv2. Step 3: The newly synthesized complementary single-stranded DNA fragments were annealed using the conditions as shown in Supplementary Table S5. Step 4: The annealed products were treated or never treated with T4 DNA ligase, and same amount of DNAs were transformed into competent *E.coli* cells. The colonies were counted, and the join sites were confirmed by DNA sequencing.

**Assembly of multiple fragments with AFEAP cloning.** To evaluate the effect of fragment number on assembly efficiency, we built a pET22b-FLAG-T4L-GGSGG_linker_-*MCM6* tandem construct, encoding T4 lysozyme (T4L)[^12^](#_ENREF_12) and MCM6 protein fused by a peptide linker, from varying number of DNA fragments (Figure 2a) with AFEAP cloning method. PCR products were subjected to AFEAP cloning protocol as mentioned above (Figure 1a). 11 unique conditions (assemblies of 2+V to 12+V fragments) were designed and tested (Figure 2a). For each test, we performed AFEAP cloning procedures as shown at Figure 1a. PCR reactions were listed in Supplementary Table S3, and thermocycling conditions were listed in the Supplementary Table S4. Here we used the assembly of 2+V fragments as example. Step 1: three PCRs were performed to generate three double-stranded DNA fragments used primer pairs 8Site13fw1 and 8Site1rv1, 8Site1fw1 and 8Site3rv1, or 8Site3fw1 and 8Site13rv1, and pET22b, the DNA sequence that encodes T4 lysozyme, or *E.coli* genome DNA as templates. PCR products were gel purified. Step 2: Two single-primer PCRs in parallel were performed to generate two complementary single-stranded DNA fragments using each purified fragment generated in the Step 1 as template and single primer of 8Site13fw2 or 8Site1rv2, 8Site1fw2 or 8Site3rv21, and 8Site3fw2 or 8Site13rv2. Step 3: The newly synthesized complementary single-stranded DNA fragments were annealed using the conditions as shown in Supplementary Table S5 to produce double-stranded DNAs with sticky ends. Step 4: The DNA fragments with complementary sticky ends were cycled ligation assembly by T4 ligase (NEB). DNA ligation reactions were performed to fuse DNA fragments in a final volume of 20 μL using T4 DNA ligase following the standard protocol from New England Biolabs. In brief, the longer and shorter DNA fragments were mixed at a molar ratio of 1:10. The reaction was incubated at room temperature for 2 hours. After heat inactivation at 65°C for 10 min, the reaction was chilled on ice. Reconstituted vector was transformed into competent *E.coli* cells. The colonies forming were counted, and the join sites can be confirmed by DNA sequencing.

**Assembly of BAC with AFEAP cloning.** A bacterial artificial chromosome (BAC), which contains 200 kb DNA sequence insert, was constructed with AFEAP cloning. PCR products were subjected to AFEAP cloning protocol as mentioned above (Figure 1a). PCR reactions were listed in Supplementary Table S3, and thermocycling conditions were listed in the Supplementary Table S4. Step 1: 9 PCRs were performed to generate 9 double-stranded DNA fragments used primer pairs BACSite1fw1 and BACSite2rv1, BACSite2fw1 and BACSite3rv1, BACSite3fw1 and BACSite4rv1, BACSite4fw1 and BACSite5rv1, BACSite5fw1 and BACSite6rv1, BACSite6fw1 and BACSite7rv1, BACSite7fw1 and BACSite8rv1, BACSite8fw1 and BACSite9rv1, or BACSite9fw1 and BACSite1rv1, and pCC1BAC^TM^ vector or genome of *Streptomyces albus subsp. albus* as templates. PCR products were gel purified. Step 2: Two single-primer PCRs in parallel were performed to generate two complementary single-stranded DNA fragments using each purified fragment generated in the Step 1 as template and single primer of BACSite1fw2 or BACSite2rv2, BACSite2fw2 or BACSite3rv2, BACSite3fw2 or BACSite4rv2, BACSite4fw2 or BACSite5rv2, BACSite5fw2 or BACSite6rv2, BACSite6fw2 or BACSite7rv2, BACSite7fw2 or BACSite8rv2, BACSite8fw2 or BACSite9rv2, or BACSite9fw2 or BACSite1rv2. Step 3: The newly synthesized complementary single-stranded DNA fragments were annealed using the conditions as shown in Supplementary Table S5 to generate double-stranded DNAs with sticky ends. Step 4: The DNA fragments with complementary sticky ends were cycled ligation assembly by T4 ligase (NEB). DNA ligation reactions were performed to fuse DNA fragments in a final volume of 20 μL using T4 DNA ligase following the standard protocol from New England Biolabs. In brief, the longer and shorter DNA fragments were mixed at a molar ratio of 1:1:1:1:1:1:1:1:10. The reaction was incubated at room temperature for 2 hours. After heat inactivation at 65 °C for 10 min, the reaction was chilled on ice. Step 5: Electroporation was carried out to transform constructed BAC into electrocompetent cells. The colonies forming were counted, and the join sites can be confirmed by DNA sequencing.

**Supplementary references**

1. Dagert, M.; Ehrlich, S. D., Prolonged incubation in calcium chloride improves the competence of Escherichia coli cells. *Gene* **1979,** *6* (1), 23-8.

2. Jin, P.; Ding, W.; Du, G.; Chen, J.; Kang, Z., DATEL: A Scarless and Sequence-Independent DNA Assembly Method Using Thermostable Exonucleases and Ligase. *ACS synthetic biology* **2016,** *5* (9), 1028-32.

3. Quan, J.; Tian, J., Circular polymerase extension cloning of complex gene libraries and pathways. *PLoS One* **2009,** *4* (7), e6441.

4. Li, M. Z.; Elledge, S. J., Harnessing homologous recombination in vitro to generate recombinant DNA via SLIC. *Nature Methods* **2007,** *4* (3), 251-6.

5. Gibson, D. G.; Young, L.; Chuang, R. Y.; Venter, J. C.; Hutchison, C. A., 3rd; Smith, H. O., Enzymatic assembly of DNA molecules up to several hundred kilobases. *Nature Methods* **2009,** *6* (5), 343-5.

6. Bitinaite, J.; Rubino, M.; Varma, K. H.; Schildkraut, I.; Vaisvila, R.; Vaiskunaite, R., USER friendly DNA engineering and cloning method by uracil excision. *Nucleic Acids Research* **2007,** *35* (6), 1992-2002.

7. Engler, C.; Gruetzner, R.; Kandzia, R.; Marillonnet, S., Golden gate shuffling: a one-pot DNA shuffling method based on type IIs restriction enzymes. *PLoS One* **2009,** *4* (5), e5553.

8. de Kok, S.; Stanton, L. H.; Slaby, T.; Durot, M.; Holmes, V. F.; Patel, K. G.; Platt, D.; Shapland, E. B.; Serber, Z.; Dean, J.; Newman, J. D.; Chandran, S. S., Rapid and reliable DNA assembly via ligase cycling reaction. *ACS synthetic biology* **2014,** *3* (2), 97-106.

9. Shao, Z.; Zhao, H., DNA assembler, an in vivo genetic method for rapid construction of biochemical pathways. *Nucleic Acids Research* **2009,** *37* (2), e16.

10. Liu, C. J.; Jiang, H.; Wu, L.; Zhu, L. Y.; Meng, E.; Zhang, D. Y., OEPR Cloning: an Efficient and Seamless Cloning Strategy for Large- and Multi-Fragments. *Scientific reports* **2017,** *7*, 44648.

11. Liang, J.; Liu, Z.; Low, X. Z.; Ang, E. L.; Zhao, H., Twin-primer non-enzymatic DNA assembly: an efficient and accurate multi-part DNA assembly method. *Nucleic Acids Research* **2017**.

12. Cherezov, V.; Rosenbaum, D. M.; Hanson, M. A.; Rasmussen, S. G.; Thian, F. S.; Kobilka, T. S.; Choi, H. J.; Kuhn, P.; Weis, W. I.; Kobilka, B. K.; Stevens, R. C., High-resolution crystal structure of an engineered human beta2-adrenergic G protein-coupled receptor. *Science* **2007,** *318* (5854), 1258-65.
