# Supplementary material for: AFEAP cloning: a precise and efficient method for large DNA sequence assembly
Source: BMC Biotechnol. 2017 Nov 14;17:81. doi: 10.1186/s12896-017-0394-x (PMC5686892; doi:10.1186/s12896-017-0394-x)
Supplement: Supplementary file 7 — PCR conditions. (DOCX 14 kb) [file 12896_2017_394_MOESM7_ESM.docx]

**Table S3** PCR conditions

|  | Reaction 1 | Reaction 2a | Reaction 2b |
| --- | --- | --- | --- |
| Template DNA | ~50 ng | ~500 ng | ~500 ng |
| Forward primer (100 µM) | 0.25 µL | 0.25 µL |  |
| Reverse primer (100 µM) | 0.25 µL |  | 0.25 µL |
| Phusion GC Buffer (5×) | 10 µL | 10 µL | 10 µL |
| dNTPs (10 mM) | 1 µL | 1 µL | 1 µL |
| DMSO (100%) | 1.5 µL | 1.5 µL | 1.5 µL |
| Phusion High Fidelity DNA  Polymerase | 1 µL | 1 µL | 1 µL |
| add water to | 50 µL | 50 µL | 50 µL |
