# Supplementary material for: AFEAP cloning: a precise and efficient method for large DNA sequence assembly
Source: BMC Biotechnol. 2017 Nov 14;17:81. doi: 10.1186/s12896-017-0394-x (PMC5686892; doi:10.1186/s12896-017-0394-x)
Supplement: Supplementary file 8 — PCR thermocycling conditions. (DOCX 13 kb) [file 12896_2017_394_MOESM8_ESM.docx]

**Table S4** PCR thermocycling conditions

| **Step** | **Temperature** | **Time** |
| --- | --- | --- |
| Initial Denaturation | 98°C | 5 min |
| 20 Cycles | 98°C | 20 seconds |
|  | 60-50 °C, step -0.5 °C | 20 seconds |
|  | 72°C | 1 minute/kbp |
|  | 98°C | 20 seconds |
| 10 Cycles | 52 °C | 20 seconds |
|  | 72°C | 1 minute/kbp |
| Final Extension | 72°C | 10 minutes |
| Hold | 4 °C |  |
