# Supplementary material for: AFEAP cloning: a precise and efficient method for large DNA sequence assembly
Source: BMC Biotechnol. 2017 Nov 14;17:81. doi: 10.1186/s12896-017-0394-x (PMC5686892; doi:10.1186/s12896-017-0394-x)
Supplement: Supplementary file 9 — PCR product reannealing conditions. (DOCX 14 kb) [file 12896_2017_394_MOESM9_ESM.docx]

**Table S5** PCR product reannealing conditions

| Steps | Temperature (°C) | Time (min) |
| --- | --- | --- |
| 1 | 95 | 5 |
| 2 | 90 | 1 |
| 3 | 80 | 1 |
| 4 | 70 | 0.5 |
| 5 | 60 | 0.5 |
| 6 | 50 | 0.5 |
| 7 | 40 | 0.5 |
| 8 | 37 | Holding |
